# A Metaheuristic Optimization Approach for Parameter Estimation in Arrhythmia Classification from Unbalanced Data

**DOI:** 10.3390/s20113139

**Published:** 2020-06-02

**Authors:** Juan Carlos Carrillo-Alarcón, Luis Alberto Morales-Rosales, Héctor Rodríguez-Rángel, Mariana Lobato-Báez, Antonio Muñoz, Ignacio Algredo-Badillo

**Affiliations:** 1 Department of Computer Science, Instituto Nacional de Astrofísica, Óptica y Electrónica (INAOE), Tonantzintla, Puebla 72840, Mexico; jccarrillo@inaoep.mx; 2 Faculty of Civil Engineering, Conacyt-Universidad Michoacana de San Nicolás de Hidalgo, Morelia 58030, Michoacán, Mexico; lamorales@conacyt.mx; 3 Technological Institute of Culiacan, Culiacan, Sinaloa 80220, Mexico; hrodriguez@itculiacan.edu.mx; 4 Higher Technological Institute of Libres, Libres, Puebla 73780, Mexico; mariana.lobato@upaep.edu.mx; 5 Engineering Department, University of Guadalajara, Av. Independencia Nacional 151, Autlán, Jalisco 48900, Mexico; jose.munoz@cucsur.udg.mx; 6 Department of Computer Science, Conacyt-Instituto Nacional de Astrofísica, Óptica y Electrónica (INAOE), Tonantzintla, Puebla 72840, Mexico

**Keywords:** electrocardiogram (ECG), signal processing, machine learning, arrhythmia, unbalanced

## Abstract

The electrocardiogram records the heart’s electrical activity and generates a significant amount of data. The analysis of these data helps us to detect diseases and disorders via heart bio-signal abnormality classification. In unbalanced-data contexts, where the classes are not equally represented, the optimization and configuration of the classification models are highly complex, reflecting on the use of computational resources. Moreover, the performance of electrocardiogram classification depends on the approach and parameter estimation to generate the model with high accuracy, sensitivity, and precision. Previous works have proposed hybrid approaches and only a few implemented parameter optimization. Instead, they generally applied an empirical tuning of parameters at a data level or an algorithm level. Hence, a scheme, including metrics of sensitivity in a higher precision and accuracy scale, deserves special attention. In this article, a metaheuristic optimization approach for parameter estimations in arrhythmia classification from unbalanced data is presented. We selected an unbalanced subset of those databases to classify eight types of arrhythmia. It is important to highlight that we combined undersampling based on the clustering method (data level) and feature selection method (algorithmic level) to tackle the unbalanced class problem. To explore parameter estimation and improve the classification for our model, we compared two metaheuristic approaches based on differential evolution and particle swarm optimization. The final results showed an accuracy of 99.95%, a F1 score of 99.88%, a sensitivity of 99.87%, a precision of 99.89%, and a specificity of 99.99%, which are high, even in the presence of unbalanced data.

## 1. Introduction

The cardiovascular diseases are a set of heart and blood vessel disorders. According to the World Health Organization, 17.9 million people die each year [1] due to cardiovascular disease. For this reason, the detection of heart bio-signal abnormalities (i.e., arrhythmias) by electrocardiograms it is vital. Arrhythmias are disturbances or irregularities of the cardiac electrical impulses [2].

An electrocardiogram (ECG) is the record of the electrical activity of the heart; this activity is detected by electrodes attached to the skin. ECG measures the electrical potentials generated by the heart and obtains information about heart rate, disorders, the position of the heart inside the body, dilation of the cavities of the heart, the origin of the propagation of the electrical activity, possible abnormalities, injuries due to insufficiency in the blood supply or produced by some agent, and heart malfunction due to dehydration or drugs [3].

**Figure 1:**
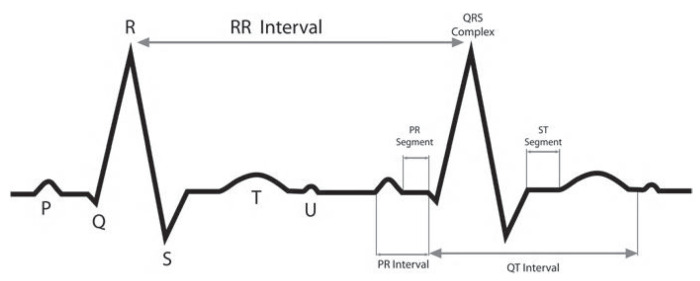
The ECG waves, segments, and intervals [4].

Physicians use ECG signals to detect cardiac problems, usually via an electrocardiograph with the 12-lead standard, allowing them to observe the heart’s activity from 12 different perspectives. The ECG signals obtained are cycles of depolarization and repolarization, where the normal beats have a rhythm called sinus, which includes the QRS complex, the P, Q, and T waves. Figure 1 shows waves and intervals in two cycles of an electrocardiogram signal.

Unbalanced data refers to a classification problem where the number of observations per class is not equally distributed. It means that the dataset has a large amount of data/observations of one or more classes (known as majority classes) and far fewer observations for one or more other classes (referred to as the minority classes). Many datasets used for the arrhythmia-beat classification have the problem of unbalanced data. The reason for unbalanced data relates to the nature of arrhythmias, since they are cardiac abnormalities that do not appear regularly. It occurs when an ECG signal is captured from a patient who is suspected of having arrhythmia from a device called Holter for monitoring the patient’s cardiac signals for several hours. During this time, the arrhythmia will probably appear for a few seconds, and in the remnant time, the signal is communicated normally. So we have arrhythmia signals that last seconds versus normal heartbeat signals that last minutes or even hours. Hence, this means that when creating an ECG dataset, it is not naturally balanced. In other words, a balanced dataset is not going to be found. Now, after the signals are captured, and the dataset is created, this imbalance problem can be mainly reduced by using two techniques: oversampling and/or undersampling; each one has advantages and disadvantages. In our case, we chose undersampling, because oversampling implies a need to validate synthetic data. Considering that we are developing applications with a health orientation, the validation is not a trivial problem because it involves a team of medical personnel specialized in cardiac signals, such as a cardiologist, validating large amounts of data. For example, in MIT-BIH Arrhythmia Database, the instances of normal beat (74,758) and left bundle branch block beat (8072), determined as the major classes, tend to surpass the numbers of cases of arrhythmia beats, such as aberrant atrial premature beats (150) and entricular escape beats (106), minority classes. Notably, this leads to a multi-class classification problem with data not equally distributed; see Table 2 in Section 3.2. Generally, this problem deals with the trade-off between recall (percent of truly positive instances that were classified as such) and precision (percent of positive classifications that are truly positive). In the context of detection, it is usually more costly to miss a positive instance than to label a negative instance falsely. In our case, we want to detect the arrhythmia cases, so we prefer F1 score as the main performance metric of the classifier model because it combines precision and recall. The unbalanced dataset problem affects the performance in most classification techniques [5], and its solution requires research on several areas; in our case, we propose a computational method using artificial intelligence. Three types of methods are used to tackle unbalanced datasets [5]:

- Data-level methods: These methods are called external methods. They consists of adding samples of the minority class—carrying out oversampling. Alternatively, there is removing samples from the majority class, known as undersampling [6].
- Algorithmic-level methods: This solution is an internal approach, wherein the goal is to design or enhance the classification algorithm. Some examples are the ensemble methods [6,7,8], feature selection methods [9,10,11], and algorithm improvement methods [12,13,14,15].
- Hybrid methods: They are combinations of data-level methods and algorithmic level methods [6,13,14,15].

In [13], a method to tackle the use of unbalanced data for classification using oversampling with the synthetic minority oversampling technique (SMOTE) was proposed, creating synthetic samples of the minority class. However, its disadvantage for ECG classification is the need for a cardiologist to validate the data created artificially. In [6], an undersampling method known as the random under-sampling technique (RUS) was proposed; in this case, some samples are removed from the majority class by a random selection. The weakness of this technique is the possibility of eliminating informative instances from the majority class.

We designed the following questions that allowed to roadmap the research presented in this paper:

- How can one design a hybrid method of parameter optimization that allows for obtaining a high-performance (F1 Score, and accuracy) arrhythmia heartbeat classification in the presence of unbalanced classes?
- What classification technique and what metaheuristic approach will allow us to obtain a high performance (F1 Score, and accuracy) arrhythmia heartbeat classification in cases of unbalanced classes?

The goal of this study was to design a hybrid parameter optimization method based on metaheuristic approaches to arrhythmia classification using unbalanced electrocardiogram signal data. Our method consists of four principal stages to carrying out this task:

1. Data acquisition: We selected an unbalanced subset of the MIT-BIH database to classify eight types of beats: four majority classes (normal beat, left bundle branch block beat, right bundle branch block beat and premature ventricular contraction) and four minority classes (aberrant atrial premature beat, Ventricular flutter wave beat, nodal escape beat, and ventricular escape beat).
2. ECG signal preprocessing: For this stage, the goals are to reduce noises and prepare the ECG signals to obtain relevant information, in the feature extraction stage, because the quality of the signals influences the performance of the classifier. We applied an amplitude normalization to reduce variations among the patients’ ECG signals and a cluster selection of instance on majority classes to reduce the number of samples.
3. Feature extraction: From the preprocessed signals, we extracted statistical features to obtain relevant information that allows the classifier to identify the label of each heartbeat.
4. Metaheuristic: In this stage, the classifier’s parameter optimization was carried out by combining the data and algorithmic levels using a metaheuristic approach:
  - Data level. The metaheuristic performs an undersampling over majority classes, selecting the instances based on clustering, and creating a new subset merging the selected instances of majority classes and all the cases of minority classes. This process allows for overcoming relevant information loss on the algorithmic level. Besides, we implemented a feature selection method to reduce high data dimensionality and improve classifier performance.
  - Algorithmic level. We use an artificial neural network as a classifier. In this case, the metaheuristic selects the number of neurons in the hidden layer, and it also selects the features from the new subset. The performance measures considered are accuracy, sensitivity or recall, specificity, precision, and F1 Score.

We establish a hypothesis that applies a hybrid optimization method to optimize the classifier parameters by combining the data and algorithmic levels based on a metaheuristic approach, which can improve on the performance of the arrhythmia heartbeat classification method up to 98% when classes in a database are unbalanced. In order to demonstrate this hypothesis, we compare two metaheuristics approaches, particle swarm optimization (PSO) [16] and differential evolution (DE) [17], to evaluate the parameter optimization performance of the classifier model. Although both metaheuristics are based on population, PSO optimizes the model obtaining information on the performance of the best individual in a population and the performance of each individual. In contrast, DE, in its version DE/rand/1/bin optimizes only with information on the performance of each individual in a population.

The paper is organized as follows: Section 2 shows related work to this research. Section 3 presents the database used and their characteristics. Then, Section 4 exposes the metaheuristic approach applied in our study and the experimental design. Section 5 presents the obtained results, and in Section 6 these are discussed. Finally, Section 7 details the conclusions of this work.

## 2. Related Work

In cardiology, the usefulness of machine learning is to provide tools with the aim of extending the capabilities of physicians, such as the improvement of identification and interpretation of disorders about heart functionality. Among the cardiovascular disorders, cardiac arrhythmias are the most common, and as a result, their precise classification has been of great interest in biomedical studies.

There are several approaches to arrhythmia classification to improve abnormality identification using electrocardiogram signals. The differences among studies are the techniques used in preprocessing, the methods employed to feature extraction and feature selection, and the proper selection of the algorithm to perform classification. For example, Chen et al. [18] proposed an approach with a combination of dynamic and projected features derived from a random projection matrix. They used a support vector machine to classify with two different approaches. The first, called the “class-based approach,” is used to classify fifteen classes, with 98.46% accuracy. The second, named the “subject-based approach,” merges the fifteen classes into five classes, according to the ANSI/AAMI EC57:1998 standard, resulting in 93.1% accuracy.

Elhaj et al. [9] developed an approach for classifying five types of arrhythmias. They extracted two types of features, linear features from coefficients of signal decomposition based on a discrete wavelet transform and non-linear features using a higher-order statistical analysis. The authors applied two classification techniques, a support vector machine with a radial base function and a feed-forward neural network with forty neurons in the hidden layer, obtaining 98.91% and 98.90% accuracy, respectively.

Hassanien et al. [10] introduced an approach to classify two types of heartbeats using a modified Pan–Tompkins algorithm (MPTA) as a feature extractor and a support vector machine (SVM) as a classifier algorithm. The authors used a metaheuristic called elephant herding optimization (EHO) to select a subset of features that improves the performance of the classifier. The metaheuristic is based on the herding behavior of the elephants used in global optimization problems. The results were 93.31% accuracy, 45.49% sensitivity, 46.45% precision, 45.48% F-measure, and 45.48% specificity.

Ashtiyani et al. [11] proposed an approach to classify three types of heartbeats using heart rate variability (HRV) and discrete wavelet transform (DWT) to extract features and to reduce the high dimensionality. The authors used a genetic algorithm to select the best features and implemented a SVM for classification. Their results were 97.14% accuracy, 97.54% sensitivity, 96.9% specificity, and 97.64% precision.

Rajesh and Dhuli [13] proposed an approach to classify five groups of heartbeats applying a nonlinear feature extraction scheme to tackle the problem of unbalanced group. They use two approaches: (1) Data level—the authors used a resampling method called “distribution based data sampling”; and (2) algorithmic level—they employed an AdaBoost classifier. Their results were 99.1% accuracy, 97.9% sensitivity, and 99.4% specificity.

Sarvan and Ozkurt [12] addressed the problem of unbalanced classes using the algorithmic level approach implementing two strategies to classify five arrhythmia types. The first strategy tackles unbalanced data using a convolutional neural network (CNN) with an increased number of epochs to improve the ability to learn of the classifier. In the second strategy, the authors used a data ensemble, including the same number of samples from each class. For creating the data ensemble, the majority classes were divided into clusters of the same size as the minority classes. In both strategies, they implemented a CNN architecture that included nine layers: three convolutional layers, three max-pooling layers, and three fully connected layers. Their results were 93.72% accuracy, 26.85% sensitivity, and 99.6% specificity.

Jiang et al. [14] introduced a multi-module neural network system (MMNNS) focused on solving the imbalance problem in ECG databases. The system has four modules: preprocessing, imbalance problem processing, feature extraction, and classification. The authors addressed the problem using a combination of the data-level approach and the algorithmic-level approach. In the data-level approach, they applied an oversampling algorithm called borderline-SMOTE (BLSM), and in the algorithmic-level approach, the authors used a module that extracts and selects features considering the bias towards to majority class. Moreover, the authors employed a CNN adopting two stages of training, the first stage with balanced data and the second stage with unbalanced data. Their system obtained 98.4% accuracy.

Gao et al. [15] introduced an approach to classify eight types of heartbeats in the case of a class imbalance tackling the problem using an algorithmic level approach. Authors used an LSTM recurrent network (LSTM: long short-term memory) with a focal loss function to optimize the weights of networks focusing on heartbeats challenging the classification. The results were 99.26% accuracy, 99.26% sensitivity, 99.14% specificity, and 99.27% of F1 score.

An interesting work was developed by Hajeb-Mohammadalipour et al. [19]. The authors used the “MIT-BIH Arrhythmia Database” where two types of classes are included: (1) beats and (2) rhythms. Authors designed a multi-sequential approach with four stages for discriminating among various malignant arrhythmias of the rhythm type. Their innovation was to integrate a method involving VF, AF, PVC, and sinus arrhythmia episode detection capabilities, by using features derived from time-frequency spectral analysis and nonlinear dynamics to discriminate VF from non-VF arrhythmias. Regarding the classification process, in the first stage to identify a type of rhythm class, they used eight-second segments, which included several beats that were not classified independently, and only identified the abnormalities in the segments of several seconds. After that, the authors classified other two types of rhythm classes but as a result of the first stage. Unlike our work, in which we identified signal segments of 0.69 s, it would be equivalent to a single beat, and we classify whether a type 8 arrhythmia is present.

As described above, the problem of unbalanced datasets affects the performance of the classifier; factors such as precision and sensitivity help to observe the impact of this issue. For example, some previously mentioned works have low values of these metrics [10,12]. The improvement of both metrics is necessary to avoid false positives and false negatives, because of arrhythmia diagnostic, if the patient is not well diagnosed, he or she could not receive treatment, or the patient could be injured by recommending the wrong medication. The contribution of our work is the performance improvement of the classifier in case of the unbalanced dataset, which could help in creating systems to help physicians in the diagnoses of arrhythmias and patient health care.

## 3. Materials and Methods

### 3.1. Multiclass Classification Methods

As a recall, we point out that supervised multiclass classification algorithms aimed at assigning a class label for each input example. In order to carry out this task, we can use two main approaches: one-versus-all (OVA) and all-versus-all (AVA); each one is described as follows [20]:

One-Versus-All (OVA): The simplest approach is to reduce the problem of classifying among *K* classes into *K* binary problems, where each problem discriminates a given class from the other K−1 classes [21]. For this approach, we require N=K binary classifiers, where the $k^{th}$ classifier is trained with positive examples belonging to class k and negative examples belonging to the other K−1 classes. When testing an unknown example, the classifier producing the maximum output is considered the winner, and this class label is assigned to that example.

The OVA approach was used by Hajeb-Mohammadalipour et al. [19]. Their approach takes one class as positive and the rest as negative, and trains the classifier. Thus, for the data having *n-classes* it trains *n classifiers*. Then, in the scoring phase all the *n-classifiers* predict the probability of a particular class and the class with highest probability is selected.

All-Versus-All (AVA): In the AVA approach, each class is compared to each other class [22,23]. A binary classifier is built to discriminate between each pair of classes, while discarding the rest of the classes. This requires building $\frac{\left(K\left(K-1\right)\right)}{2}$ binary classifiers. When testing a new example, a vote is performed among the classifiers and the class with the maximum number of votes wins.

In our case we consider the AVA approach with binary pairs of classes, and train the classifier on a subset of data containing those classes. Thus, we train a total of $\frac{\left(k\left(k-1\right)\right)}{2}$ classes. During the classification phases, each classifier predicts one class. (This is in contrast to OVA where each classifier predicts probability). Additionally, the class which has been predicted most is the answer.

In this sense, we can describe some differences between these two approaches:

- OVA has a shorter training stage than AVA and hence is faster overall, so OVA is usually preferred.
- Every single classifier in AVA uses a subset of data, so a single classifier is faster for AVA.
- AVA is less predisposed to be imbalanced in a dataset (dominance of particular classes)

Two main inconsistencies can be found using these two approaches:

- What happens if two or more classes are equally voted in the AVA approach?
- What happens if the probability is relatively close or equal in the case of OVA?

As we can see, both approaches have advantages and disadvantages. Besides, we perceive that an OVA approach is as accurate as any other based on the assumption that the classes are “independent.” Thus, the classes do not belong to a natural hierarchy, and we do not necessarily expect examples from class “A” to be closer to those in class “B” than those in class “C.” In the latter situation, especially when a few examples were available, we might suspect that an algorithm that exploited the relationships between classes could offer superior performance, leading to an interesting open question. Hence, we explore the AVA approach due to the assumption that arrhythmia classes are closer, as we showed in Figure 12.

### 3.2. Materials

For the purposes of this work, we used the ECG data gathered in the MIT-BIH Arrhythmia dataset in order to carry out a comparison among some works described in the state-of-the-art. The MIT-BIH Arrhythmia database is described as follows:

MIT-BIH Arrhythmia Database [24]: This database was downloaded from the Physionet platform, and it contains 48 half-hour excerpts of two-channel ambulatory ECG recordings obtained from 47 subjects. The subjects were 25 men and 22 women; the men were 32–89 years old and the women were 23–89 years old. The frequency of each recording is 360 Hz, with an 11-bit resolution over a 10mV range. The database has seventeen types of heartbeats and fourteen types of rhythms. From the database, we select the most typical elements (four majority classes) and the less-typical elements (four minority classes), to represent an unbalanced behavior. In Table 1, the classes are the annotations or acronyms utilized in the database to represent the name of arrhythmia, and the samples are from the limb lead II (MLII). Besides, Table 1 shows the imbalanced nature of classes because the number of samples is not the same. The sizes of majority classes (N, L, R, and V) are several times greater than those of the minority classes (!, j, a, and E). Therefore, the selected subset shown in Table 1 helps us to find ways to get better performance with unbalanced ECG Data.

We use the imbalance ratio (IR) to measure the class imbalance; we apply the Equation (1) to calculate the metric where $N_{-}$ is the number of instances in the majority class and $N_{+}$ is the number of instances in the minority class.

(1) $$IR=\frac{N_{-}}{N_{+}}$$

There is no rule about a value of IR to establish a dataset as unbalanced, but Lango [25] states that a value of IR higher than 1.5 is considered an unbalanced dataset. Tsai et al. [26] mentioned that an IR higher than ten makes it challenging to build an effective classifier, and an IR higher than 100 can be drastic so that it may deteriorate the performance of classification. Table 2 shows values of IR between classes selected from the MIT-BIH Arrhythmia Database. IR values display a severe imbalance among N class and minority classes. Besides, there is a substantial imbalance among the other majority classes and minority classes. redIn this work, we are focusing on decreasing the IR between the classes selected by carrying out undersampling over majority classes; see Tables 7 and 8 in Section 5.

## 4. Metaheuristic Approach

**Figure 2:**
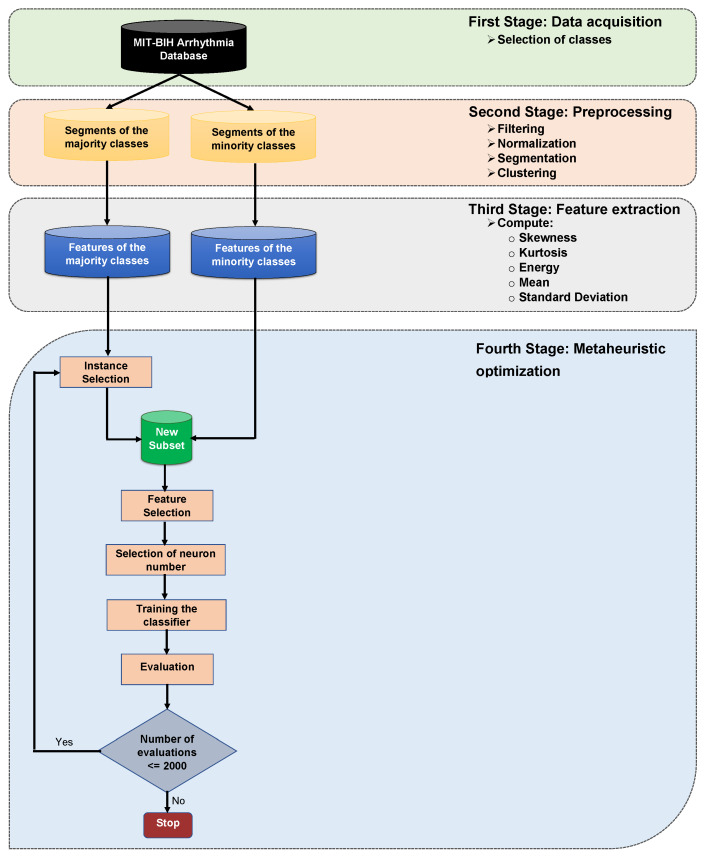
Metahuristic approach diagram.

The metaheuristic approach for heartbeat classification with unbalance data shown in Figure 2 has four stages. The first one, data acquisition, uses data from the MIT-BIH arrhythmia database for analyzing the classes to generate an imbalance context of four majority classes and four minority classes. The unbalanced ratio of the new subset of the eight classes is calculated, explained in Section 3.2. The second one, the ECG signal preprocessing stage, has four steps (see Section 4.1). First, we carry out filtering on the records to remove noises from signals. Then, an amplitude normalization to each signal is implemented to decrease the variation among ECG signals of patients. After that, the filtered signals are segmented in heartbeats. At last, the clustering method is applied to the majority classes, dividing them into smaller groups. The feature extraction is the third stage, where statistical features are extracted per heartbeat of all the signals selected; see Section 4.3. In the final stage, metaheuristic optimization, a hybrid approach to tackle the imbalance problem is applied; see Section 4.4. The metaheuristic has the aim of selecting several parameters to improve the performance of the classifier. On the data level, it selects the size of SOM features maps and the number of instances (percentage) of each majority class cluster. On the algorithmic level, the metaheuristic selects the number of neurons in the hidden layer of the artificial neural network, and the features to train and test the classifier. These parameters are represented in a vector, and a fitness function evaluates the performance obtained by each vector. In the next sections, we explain in more detail every stage.

### 4.1. Preprocessing

**Figure 3:**
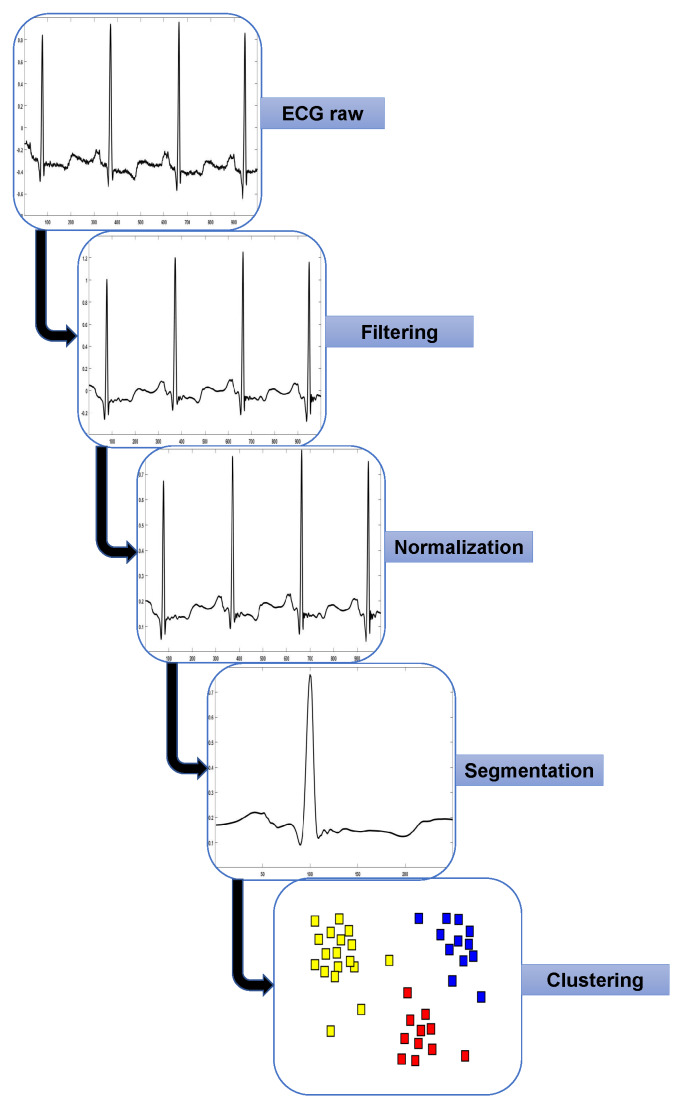
Diagram of preprocessing.

The electrocardiogram signals have several types of noise. Hence, the preprocessing stage aims to reduce the contamination (noise) caused by muscle noise, power line interference, motion artifact, and baseline drift, among other things. The preprocessing is essential because of the signal condition of ECG influences classification performance. Figure 3 shows the major steps in the preprocessing stage.

#### 4.1.1. Denoising

We select denoising as the first step of preprocessing to remove noise and to avoid edge effect at the ends of segments of the signal [27]. It is necessary to carry out filtering of the signal to delete power line interference at 50 or 60 Hz, baseline wander or baseline drift around 0.5 Hz, electromyogram noises at frequencies higher 50 Hz, and low and high-frequency noise components, which interfere with the signal analysis [28]. The principal components of electrocardiogram signals are P wave, QRS complex, and T Wave, where their frequencies are from 5 to 40 Hz [29].

We compare two independent filtering methods in two different scenarios; see Section 4.5.2. The first filtering method is a combination of low-pass and high-pass filtering. The second method is based on wavelets. The aim of the comparison is to observe the filtering process impact on the performance of the classifier model using combinations among filtering methods and metaheuristics approaches. We select the low-pass and high-pass filtering method because these types of filters have presented high performance at removing noise in electrocardiogram signals, such as muscle noise, baseline wander, power line interference, electromyography noise, and electrosurgical noise [30,31].

We used the wavelet filtering method because it has demonstrated itself to be a useful tool for analyzing non-stationary signals, such as ECG; therefore, we implemented a denoising process based on the discrete wavelet transform (DWT), as other authors did [9,32].

Here we describe the steps of both methods:

1. Low and high pass filtering method: Initially, we apply a high-pass filter with a cutoff frequency 1 Hz to remove noise of low frequencies; for example, baseline wander. Then, we use a low-pass Butterworth filter of sixth order, with a cutoff frequency of 30 Hz to delete noise of high frequencies.
2. Wavelet filtering method: We apply an interval-dependent denoising method to the ECG signals to remove noises such as power line interference, electromyogram noise, and high-frequency noise components. First, we use the Daubechies D6 (db6) wavelet basis to nine levels of decomposition. Then, we reconstruct the decomposed signal and apply a new decomposition of the reconstructed signal in nine levels using the db6 wavelet basis, removing the ninth level approximation sub-band to remove baseline wander noise. Finally, we reconstruct the signal using detailed components from one to nine sub-bands to obtain an ECG signal that is denoised and smoothed.

**Figure 4:**
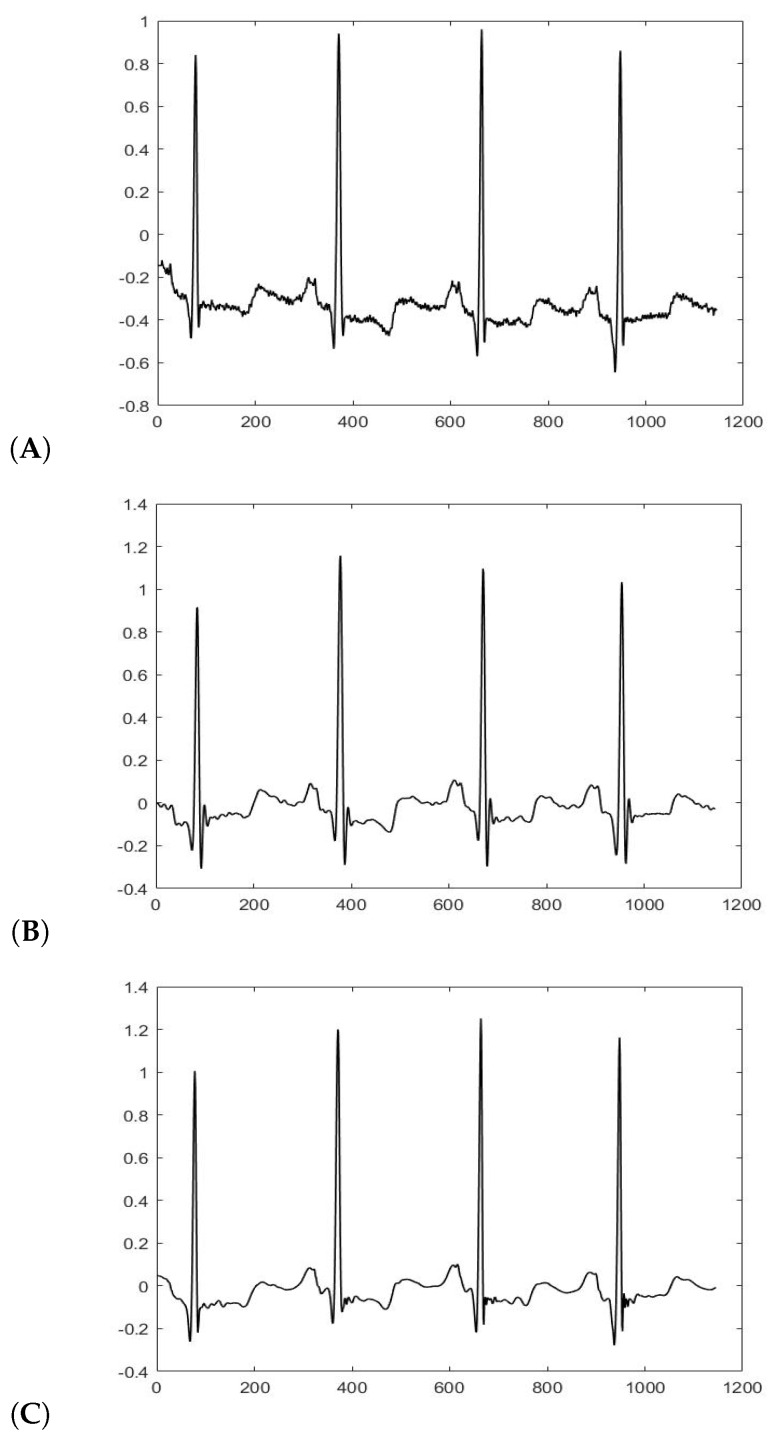
(**A**) Signal before the filtering. (**B**) Signal after the combination of high-pass and low-pass filtering. (**C**) Signal after the wavelet filtering process.

Figure 4 shows the same signal before the filtering process and after the filtering process. The raw signal in Panel A shows two types of noise, power line interference and baseline wander. Panel B shows the signal after the low-pass and high-pass filtering process; the noise of the raw signal was removed, but we can observe slight changes in amplitude: in the raw signal, the Q wave is deeper than S wave, but in signal filtered, it is the opposite. Panel C shows thhe signal after the wavelet filtering process; the power line interference and baseline wander were removed, and the signal is smoother than signals in Panel A and Panel B.

#### 4.1.2. Normalization

**Figure 5:**
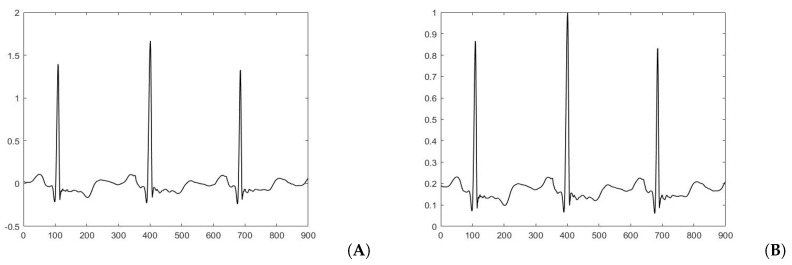
(**A**) Signal before normalization. (**B**) Signal after normalization.

Amplitude normalization has been used as one step in the preprocessing stage by authors such as Zadeh et al. [33], to eliminate the effect of amplitude changes. In contrast, Donoso et al. [34] mentioned that amplitude normalization is optional but helps to compare the signals from different patients visually. Thomas et al. [35] applied a normalization process to reduce the amplitude variance from file to file. In this work, we normalize ECG signals into a range from –1 and 1 to reduce variations among patients’ signals, using Equation (2), proposed by Kutlu et al. [36], where Y(s) is the normalized data matrix and X(s) is the sth sample vector. Figure 5 shows signals before (Panel A) and after (Panel B) normalization; both are visually similar, but their scales of amplitude are different.

(2) $$Y\left(s\right)=2\cdot \left(\frac{X\left(s\right)-X{\left(s\right)}_{min}}{X{\left(s\right)}_{max}-X{\left(s\right)}_{min}}\right)-1$$

#### 4.1.3. Segmentation

**Figure 6:**
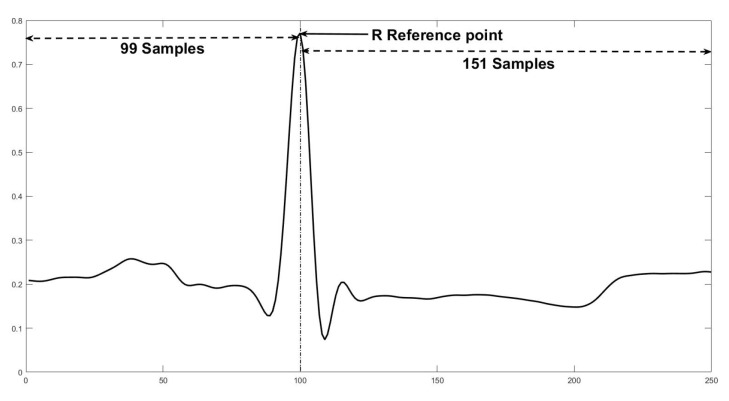
Heartbeat segmentation example.

Sixty bpm (beats per minute) is a normal heart rate for a healthy adult [30]. There is not a rule about the number of sampling points in each segment, but Chen et al. [18] recommended 300 sampling points and Elhaj et al. [9] suggested 200 sampling points. We chose 250 sampling points, resulting in a segment equivalent to 0.69 seconds, which includes P wave, QRS complex, and T wave. To carry out the segmentation, we first locate the heartbeat annotation. Then, we use the QRS complex location like a reference point, and we select 99 sampling points from the left side and 151 sampling points from the right side in the ECG signal; Figure 6 shows an example.

### 4.2. Clustering

In our work, this task is a crucial step because it is carried out an undersampling. There are several undersampling methods, such as [6,26,37,38,39,40]. In our study, we focus on preserving informative samples when the size of the majority classes is reduced based on clustering, such as in [26,38].

A clustering process is used to organize the signal segments in groups (clusters) with similar features based on a similarity degree. We use self-organizing maps because the neural network preserves input space topological properties. Through the mapping, the relative distance among the points is preserved, allowing clustering the data without knowing the class memberships of the input data.

#### 4.2.1. Self Organizing Map

**Figure 7:**
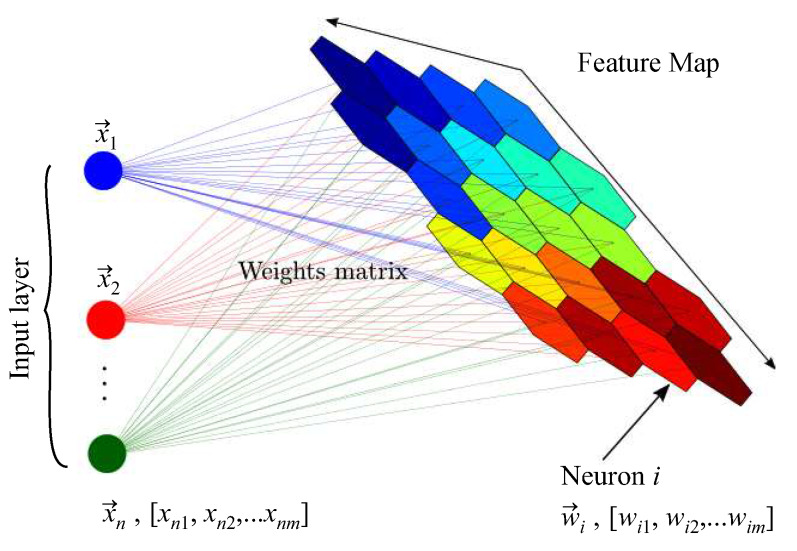
Self-organizing map structure [42].

A self-organizing map (SOM) is a neural-network algorithm based on unsupervised learning. Its objective is to find the optimal position of the cluster centers from data through an iterative learning process. A SOM process has four principal stages: initialization, competition, cooperation, and adaptation. In the initialization, weighted vectors are initialized with random values. In competition, for each instance, the neurons compute values of a discriminant function, and the neuron with small values wins. In cooperation, the winner neuron determines a neighborhood of excited neurons. In adaptation, the neuron winner is enhanced by the reduction of discriminant function values of the excited neurons through adjustment weights [41]. Figure 7 shows a SOM structure, where ${\vec{X}}_{n}$ is the vector of features, ${\vec{W}}_{i}$ is the weight vector of the neuron, and the neuron group is a feature map.

#### 4.2.2. Clustering Process

**Figure 8:**
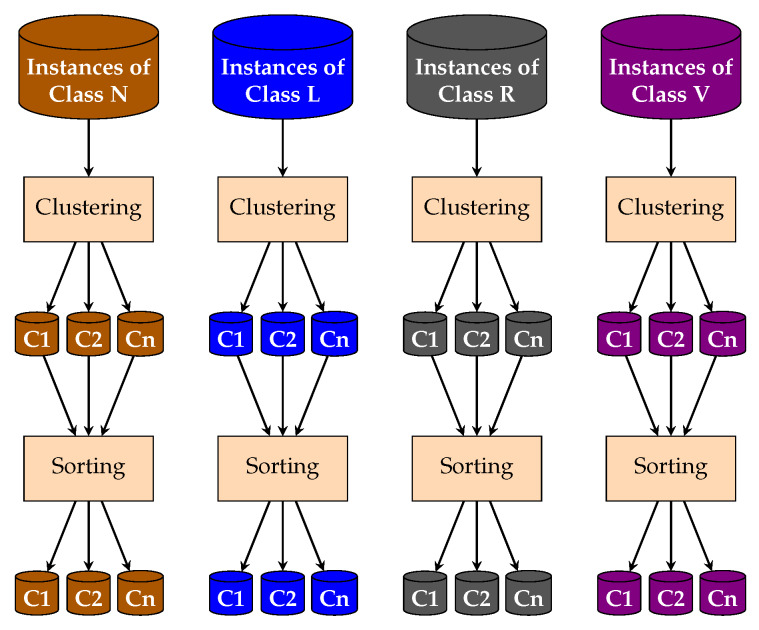
Clustering processes of majority classes.

Clusters are used in the fourth stage of the methodology for the instance selection to undersample the majority classes. Figure 8 shows a diagram of the clustering process using SOM. In the first step, we select the size of a two-dimensional feature map, choosing the number of rows and columns to form a grid, where each node in the grid is a neuron. The total number of neurons is equal to the number of rows multiplied by the number of columns. We test the feature map size of the same number the rows and columns, from two to ten, 2 × 2, 3 × 3, ..., 10 × 10. This process is carried out to observe the performance of the classifier using different configurations. Then the network is trained, and once it finished, the number of obtained clusters is equal or smaller than the number of neurons. In SOM, the weight vector associated with each neuron is the center of each cluster. Hence, each cluster computes the euclidian distance from its instances to its center. This allows us to know the farthest instances to the center and consider them like “noisy instances”. Finally, the instances of each cluster are sorted in ascending order. Besides, the undersampling is carried out by the metaheuristic, selecting a percentage of the instances closer to each cluster’s center.

### 4.3. Feature Extraction

The feature extraction process is essential to classifying ECG signals due to information gathering to differentiate among heartbeats of arrhythmias. The statistical features are extracted because, through them, the type of distribution is analyzed, as is the level of complexity that ECG signals exhibit; this allows discriminating variations among heartbeats [30]. Several authors report the use of statistical features [7,9,43,44,45].

**Figure 9:**
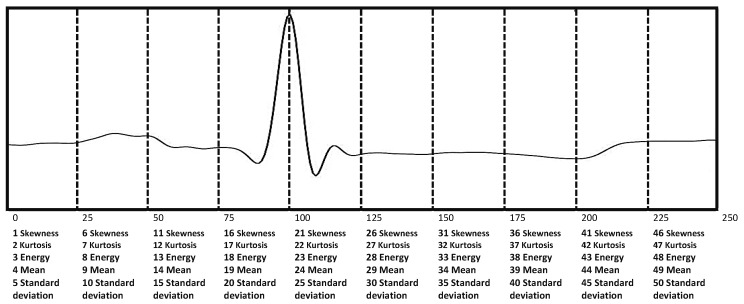
Feature extraction from segments.

The first step in feature extraction is to divide the heartbeat into subsegments. Since each heartbeat has 250 samples—see Section 4.1.3 of segmentation—we divide it into subsegments with equal numbers of samples. For each subsegment, five statistical characteristics are extracted. The subsegment division can be made through several division options; for example, 5 subsegments of 50 samples (obtaining 25 features), 10 subsegments of 25 samples (obtaining 50 features), 25 subsegments of 10 samples (obtaining 125 features), and 50 subsegments of 5 samples (obtaining 250 features), among others. We recognize that from 25 subsegments, the dimensionality begins to increase. Thus, the two most viable options are subsegments of size 5 or subsegments of size 10. In order to choose between these two options, some tests were carried out; the results are shown in Table 3. We observed that 10 subsegments (50 characteristics) demonstrate a higher performance regarding the F1 Score mean and a smaller standard deviation than when only 5 subsegments (25 characteristics) are used. Hence, Figure 9 shows the segment division and the order of each feature. For each segment, the extracted statistical attributes are skewness, kurtosis, energy, mean, and standard deviation. In the final of the process, the number of features is fifty.

### 4.4. Metaheuristic Optimization

In this stage, we propose a hybrid optimization method to optimize the classifier’s parameters through combining the data and algorithmic levels based on a metaheuristic approach to tackle the unbalanced class problem. We compare two metaheuristics approaches, particle swarm optimization (PSO) [16] and differential evolution (DE) [17], in similar conditions to search for the combination of parameters improving the performance of the classifier.

#### 4.4.1. Particle Swarm Optimization

Particle swarm optimization (PSO) is a metaheuristic for optimization based on bird and fish flock movement behavior. In the standard PSO algorithm, a swarm is formed by particles that change position; each particle is a potential solution to the problem, and they change position according to three principles: (1) to maintain inertia, (2) to change the conditions according to its optimal position, and (3) to change the conditions according to the swarm’s optimal position [16].

#### 4.4.2. Differential Evolution

Differential evolution (DE) is a stochastic algorithm based on the population. It optimizes real-valued functions through the iterative execution of four steps: initialization, mutation, crossover, and selection [17]. Each step is described as follows:

- Initialization: In this step, a population of parameter vectors is generated randomly between lower bound and upper bound.
- Mutation: In each generation or iteration, individuals of the current population become target vectors. For each target vector, the algorithm randomly selects from the population, three different vectors to the current target vector; then, a donor vector is created from the weighted difference of two vectors to the third.
- Crossover: After generating the donor vector, the recombination operation or crossover is performed to enhance the potential diversity of the population. In this step are randomly selected elements from the current target vector and elements of the donor vector that are merged to create a trial vector.
- Selection: Finally, the performances of the current target vector and trial vector are computed with a fitness function; the values obtained are compared; and the vector with the highest function value continues to the next generation. The others are removed.

#### 4.4.3. Artificial Neural Networks

An artificial neural network (ANN) is a mathematical model inspired by a biological neural network [46]. The model includes artificial neurons interconnected; each interconnection is associated with adjustable weights. The neurons are organized in layers, one input layer, one output layer, and one or several hidden layers. The ANNs try to solve linear and non-linear classification problems with different network structures and the types of learning (supervised or unsupervised).

#### 4.4.4. Metaheuristics Optimization for Classification Model

We compare the performances of two metaheuristics, particle swarm optimization (PSO) [16] and differential evolution (DE) [17], to search the combination of instances, features, and a number of neurons that allow us to obtain higher performance in the classification. In both metaheuristics, each combination is represented as a vector of real numbers. Table 4 shows the vector structure. The first element is to select the self organizing map (SOM) size from 2 to 10. The elements from 2 to 5 are used to select the percentage of the instances selection (undersampling). The lower bound of elements 2, 4, and 5 is 0.01, and for element 3 it is 0.001. The upper bound of the four elements is 0.999. Element 6 is for selecting the number of neurons, from 10 to 500. Finally, the elements from 7 to 56 are used for selecting features. A value of 1 means the feature is selected, and a value of 0 the feature is not being chosen. In elements 1, 6, and 7–56, integer numbers are required, and for this reason, their real values are rounded.

Algorithm 1 shows the classification process; it starts when the metaheuristics evaluate a representation vector (see Table 4) through the objective function. In the next list, we explain the steps of the objective function:

1. First, in line 1 we load the clusters according to parameter one of the representation vector (SOM feature map size). Then, from lines 3 to 10, the sizes of majority classes are reduced (undersampling), selecting instances from the loaded clusters. The numbers of instances to select for each cluster are the parameters in the elements from 2 to 5 of the vector.
2. When completing instance selection, in line 11, a new subset is formed by merging instances selected of majority classes and all instances of minority classes.
3. In line 12, a new vector is obtained from elements 7–56 of the representation vector. From lines 13 to 15, we iterate over the elements of the new vector, and if the value of the element is 1, the feature is selected or removed if its value is 0. Hence, the new subset has fewer features.
4. We use k-fold cross-validation. In line 16, we set the value of k = 10. Line 17 divides the new subset into ten folds. From lines 18 to 23, we iterate over each fold, taking each fold as a test dataset and the remaining folds as the training dataset. In each training of the artificial neural network, the number of neurons in the hidden layers is the selected parameter by the metaheuristic at the sixth element of the vector.
5. Finally, in line 24, we compute the average performance over the ten folds. Line 25 returns the F1 score value indicating the performance of the model.

In the metaheuristics, each evaluation is a call to the objective function. It returns the F1 score, and the metaheuristics try to maximize the value of this metric. When the number of calls to the objective function is greater than 2000, the metaheuristics stop the exploration.
**Algorithm 1:** Objective function.    **Data:** Instance clusters of majority classes.    **Data:** Instances of minority classes.    **Output:** F1 Score value.    **Input:** Representation Vector
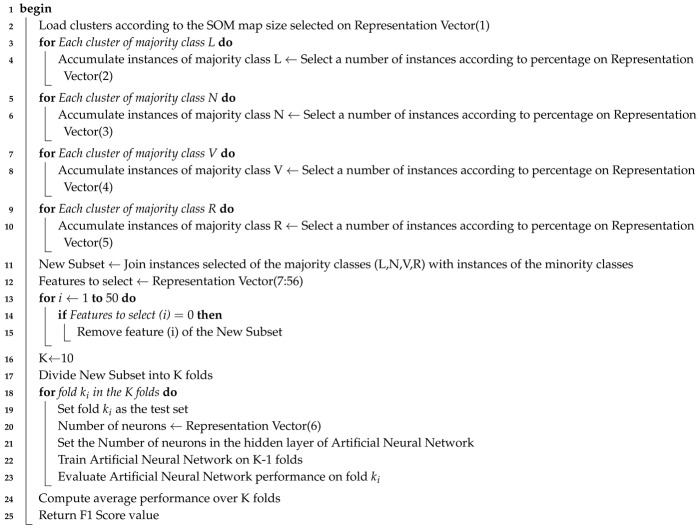


### 4.5. Cases of Experimentation

#### 4.5.1. Performance Measurements

We use five metrics to evaluate the performance of the classification accuracy (Acc), sensitivity or recall (Sen), specificity (Spe), precision (Pre), and F1 score (F1), where TP is true positive, FN is false negative, TN is true negative, and FP is false positive. The equations for each metric are defined as follows:

(3) $$Acc=\frac{TP+TN}{\left(TP+TN+FP+FN\right)}\cdot 100$$

(4) $$Sen=\frac{TP}{\left(TP+FN\right)}\cdot 100$$

(5) $$Spe=\frac{TN}{\left(TN+FP\right)}\cdot 100$$

(6) $$Pre=\frac{TP}{\left(TP+FP\right)}\cdot 100$$

(7) $$F1Score=2\cdot \frac{precision\cdot sensitivity}{precision+sensitivity}\cdot 100$$

The metrics to each class are calculated with the above equations using a macro averaged approach [47]. The overall metric is computed with the mean of the metric results of all classes. We choose accuracy as a measure of the overall performance of the classifier model. Sensitivity is a metric related to the rate of false negatives. Precision is a measure linked to the rate of false positives. Specificity is a metric to determine whether a prediction does not belong to a class. F1 score as the harmonic mean of precision and sensitivity, taking both metrics into account to estimate the performance of the classifier model when there are unbalanced class problems.

#### 4.5.2. Experiments Configuration

**Figure 10:**
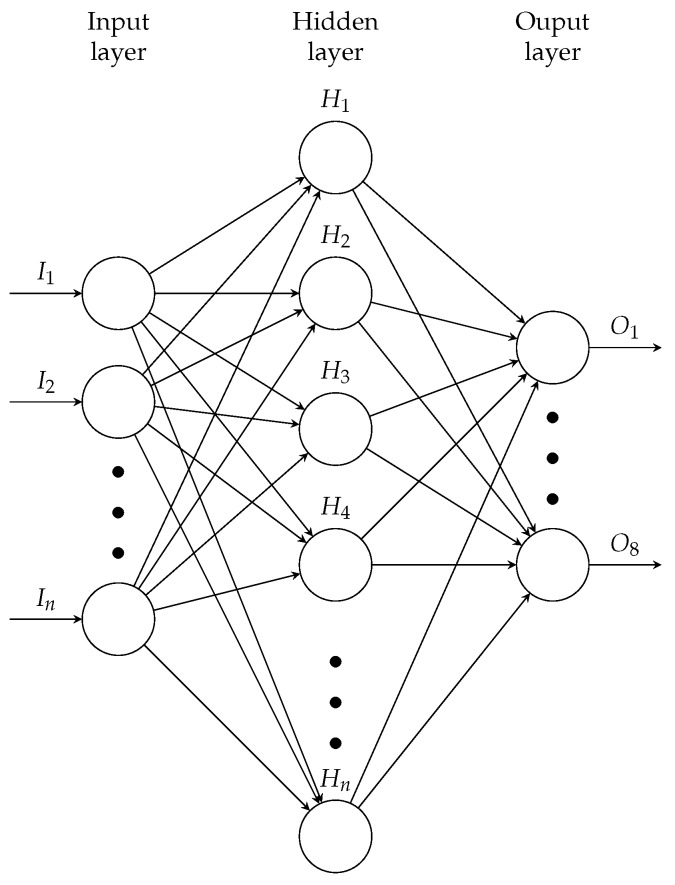
Artificial neural network structure.

We perform four experiments to measure the optimization of the classification process. The experiments have two main goals: (1) to prove two configurations for each metaheuristic (PSO and DE) changing their type of filtering method (HL and wavelets), and (2) to evaluate their overall performance. Table 5 shows the configuration of the four experiments where the number of particles (PSO) or parameter vectors (DE) is 50, and the maximum number of evaluations is 2000; we select this number to maintain comparable scenarios among the four experiments in similar conditions. Therefore, we can observe the performance of metaheuristics in a controlled context. The structure of the ANN used in all experiments is: (1) a number of neurons in the input layer equivalent to the number of features selected by metaheuristic, (2) eight neurons in the output layer accordingly with the number of classes, (3) the number of neurons in the hidden layer is selected by metaheuristic, and (4) the learning algorithm based on the scaled conjugate gradient, which updates the weights and the bias; this is illustrated in Figure 10.

In four experiments, the bounds are the same; it means that a particle (PSO) or a parameter vector (DE) has the same structure; see Table 4. The map size indicates the configuration of the feature map in SOM. The metaheuristic has the ability to select eight configurations, and the number of clusters increases in larger map sizes. The percentage of instances to select is the same in the majority class, except in the lower bound of the Class N, which is lower than the other three classes because the number of samples is greater than the other classes. The metaheuristic can select 490 options of the number of neurons in the hidden layer to improve the performance of the classifier. Figure 9 shows the number of fifty features that the metaheuristic can select according to two options: when the value is 1, the feature is selected, and it is rejected for a value of 0. Moreover, we repeat the four experiments ten times to observe the convergence of the algorithms and the differences in each repetition.

In experiments 1 and 3, the metaheuristic was PSO. In both experiments, parameter *w* was used to control the velocity of the particles, related to the control of exploration and exploitation; $w_{max}$ is the searching ability for the global (exploration), and $w_{min}$ is the searching ability for the local (exploitation). Accelerated constants in PSO are the stochastic acceleration weight of particles $c_{1}$ toward the personal best of each particle, and $c_{2}$ toward the global best of the population. We use typical values for the parameters $w_{max}$ of 0.9, $w_{min}$ of 0.4, $c_{1}$, and $c_{2}$ equal to 2.

In experiments 2 and 4, the metaheuristic was DE. We use the strategy DE/rand/1/bin, where “rand” means that parameter vectors selected for the mutation are chosen at random, “1” is the number of solution couples, and “bin” indicates that the recombination is binomial. Mutation factor “F” is a control parameter in the mutation stage; it manages the ability for exploitation and exploration. In the recombination stage of DE, the crossover rate “Cr” is a control parameter in the binomial crossover where higher values imply a lesser influence of mutant vector on the trial vector. The typical values used in the two experiments were F = 0.5 and Cr = 0.9.

## 5. Results

In the diagnosis of a patient, it is essential to avoid false positives (FP) and false negatives (FN) because an incorrect diagnosis implies that the patient may suffer an injury by recommending the wrong medication. FP and FN are crucial in our work. Therefore, we use the F1 score as the main performance metric of the classifier model; it is a suitable metric when there are unbalanced classes.

**Figure 11:**
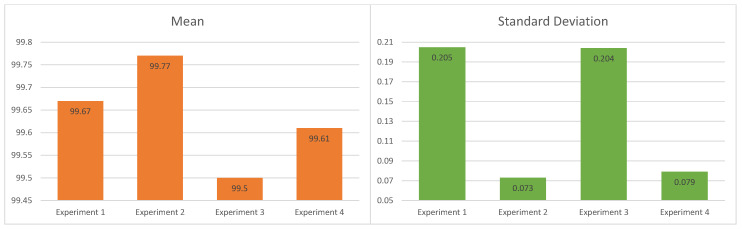
Means and standard deviations of ten tests on the four experiments.

Table 6 shows the results of the four experiments in ten tests, and Figure 11 shows the mean and the standard deviation of the F1 score in each experiment. Experiment 2 obtained the best results of the four experiments due to the fact that the value of the overall mean is 99.77%, the highest. The configuration of the experiment uses a combination of the low-pass and high-pass filtering method with a differential evolution (DE) algorithm. The standard deviation value is 0.073%, the lowest, due to a low dispersion of the results. Experiment two is more precise and repeatable by the low value of the standard deviation, and more accurate than the other three experiments by the high value of the mean. In the next list, we explain the observations in the other experiments:

- Experiment 1: The mean is 99.67%. That is the second-highest performance of the four experiments—0.10% lower than the second experiment. The disadvantage is a 0.132% higher standard deviation than experiment 2. For this reason, the first experiment is less repeatable compared to the second experiment.
- Experiment 3: It has a value of the mean of 99.50%; this performance is the lowest of the four experiments. Moreover, the experiment is less repeatable compared with experiment 2 because its standard deviation is the second-highest of four experiments with a value of 0.204%.
- Experiment 4: It obtains 99.61% for the mean; that is the second-lowest performance of the four experiments; its advantage is that its standard deviation is of 0.079%—0.006% higher than the second experiment.

In this work, the best performance was obtained by the combination of a low and high-pass filtering method. This method was applied in experiment 1 and experiment 2. The F1 scores were 99.67% and 99.77% respectively, against the wavelet filtering method in experiments 3 and 4 with means of 99.50% and 99.61%; therefore, the selection of the filtering method is fundamental in the preprocessing stage of the methodology because of its impact on the classifier’s performance.

We compare the metaheuristics (PSO and DE) in similar conditions, observing that DE had a higher performance than PSO. In experiments 1 and 2, the combined filtering (low-and-high pass) is used. In the second experiment, DE had a mean of 99.77%—higher than the mean of 99.67% obtained by PSO in the first experiment. The wavelet filtering method is applied for experiments 3 and 4. DE had a higher performance again with a mean value of 99.61% in the fourth experiment versus a mean of 99.50% of the third experiment (PSO). The experiments 2 and 4, with DE, had less variability; their values of standard deviation were 0.073% and 0.079%, respectively. Against higher values of PSO in experiments 1 and 3 (0.205% and 0.204%), this behavior occurred due to the fact that PSO tended to get trapped at local maximums. The maximum values are shown in bold.

Table 7 shows the best solutions of the four experiments. From the five computed metrics, we observe that experiments 1 and 2 obtained the best performances with values of F1 score of 99.93% and 99.88%, respectively. In the resulting model in the four trials, the selected parameters by the metaheuristics were different for each model. The first parameter chosen by the metaheuristic is the size of the SOM feature map; see Section 4.2.2, showing for each configuration that the number of clusters was different. For example, when the map size is 2 × 2, the maximum number of clusters is four; this means that the majority class is divided into four smaller groups. We observe that metaheuristic tends to select smaller map sizes. In the experiments, the map sizes of the best solutions are 2 × 2 and 4 × 4; this means that the best performances will be gotten whether the majority classes are divided into a low number of clusters.

The total number of instances used in each experiment is shown in the row called “Size of the new subset”. The size of the majority classes (L, N, V, and R) has been reduced as a consequence of the undersampling; see Table 7. All the instances of the minority classes were conserved for all the experiments. We observed in Table 7 that the final size reduction in the new subsets was higher than 90% in the four experiments in comparison with the original set, which had 98,165 instances. For example, we observed that for the class R, the number of instances contained in the original dataset were 7255. Nevertheless, in experiment 1 we used only 2167; for the second 1802; the third selected 1887; and the fourth experiment used 2463. The original instances for each arrhythmia are described in Table 1, and the reduction of the number of instances for all the majority classes is shown in Table 7. Thus, we can conclude that applying grouping-based undersampling helps improve classifier performance.

The parameters selected for “number of neurons” and “features selected” were different in the four experiments. Therefore, a metaheuristic approach is vital to improving the performance of the artificial neural network, because it optimizes the parameters according to the subset of the selected instances. Figure 9 presents the selected features showed in Table 7; notice that the numbers of features selected by PSO in experiments 1 and 3 are lower (23 and 27) than the numbers of features selected by DE in experiments 2 and 4 (44 and 43).

Table shows the scatter plot of the class instances considered in this work. Plot A displays the full dataset that describes the eight arrhythmia types. As we can observe, the multiclass classification problem is not trivial due to the overlapping between several arrhythmias. We obtain the plots B–E by applying the clustering and featuring extraction steps. A critical remark is that we still have unbalanced classes, but their overlapping decrease allows for build better frontiers between the classes to make them separable.

**Figure 12:**
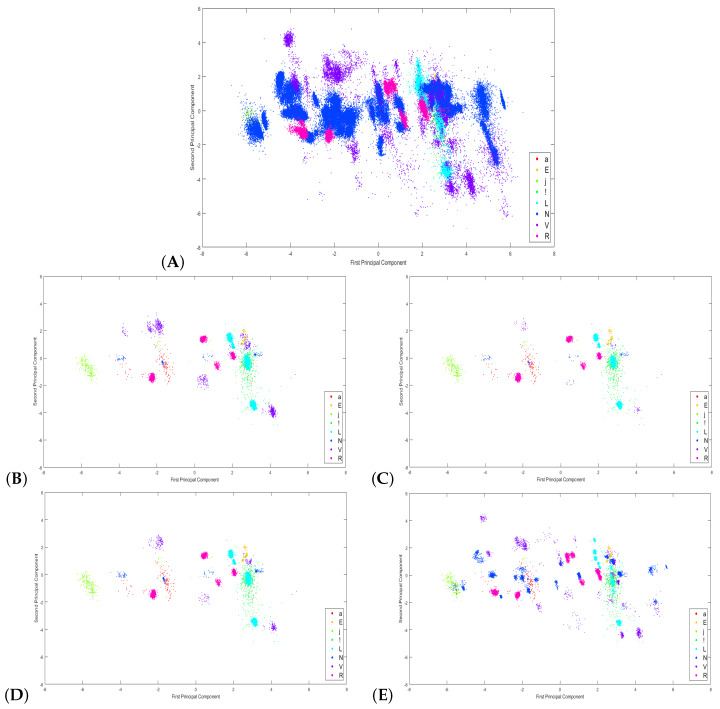
(**A**) Scatter plot of instances on the full dataset. (**B**) Scatter plot of instances on the experiment 1 dataset. (**C**) Scatter plot of instances on the experiment 2 dataset. (**D)** Scatter plot of instances on the experiment 3 dataset. (**E**) Scatter plot of instances on the experiment 4 dataset.

Figure 12 shows the scatter plots of the original set and the new subsets for each experiment. These plots were obtained by applying principal component analysis (PCA) to each of the beats to reduce the number of dimensions from 250 to just two. Therefore, from the components resulting from the analysis of each heartbeat, the first two components were selected to display the plot in two dimensions. With these plots, we are able to observe the impact generated by the subsampling. Plot A displays the full dataset that describes the eight arrhythmia types. As we can observe, the multiclass classification problem is not trivial due to the overlapping between the majority classes over the minority classes; for example, the N and V classes. We obtain the plots of B–E by applying undersampling, clustering, and feature extraction steps. In the plots of experiments 1–3 (Panels B, C, and D), we observe a slightly similar distribution of the instances due to the three experiments having implemented the same cluster configuration (See Table 7, “ SOM map size”). In Panel E, we obtain a different distribution of the instances; this is a consequence of a higher number of clusters used, so a more significant overlap is observed between them. A critical remark is that we still have unbalanced classes, but their overlapping decreases, allowing us to build better frontiers between the classes to make them separable.

### 5.1. Analysis of the Classification Model

In Table 8, we describe the “imbalance ratio” (IR) to measure the degree of imbalance between the arrhythmia classes. As a recall, if the value of IR is one, it means that the classes are balanced, implying that they have the same number of instances. When the IR value is different from 1 denotes a degree of inequality. Particularly, Table 8 shows a comparison between the IRs of the original set and the subset used in experiment 2 due to representing our best performance.

We observed a decrease in the IR values of the subset of experiment 2, meaning that the unbalance between the majority classes and minorities decreased. Since no IR value is equal to 1, it can be inferred that the classes are not balanced. Nevertheless, the imbalance reduction is significant as it enables the classifier to achieve better overall performance and improve their learning of minority classes without the need to generate synthetic data. Besides, we show in Table 7 that the proposed approach allows obtaining a high performance despite the fact that the new subset still has a certain degree of imbalance.

Table 9 shows the confusion matrix used to evaluate the performance of the best solution in experiment 2. From the confusion matrix are extracted true positives (TP), false positives (FP), true negatives (TN), and false negatives (FN) to calculate the values of the metrics. In Section 4.5.1, we show equations used to compute the performance of each class and explain how to calculate the overall metrics.

The classifier’s performance is measured by using the five metrics determined in Section 4.5.1. First, it is necessary to calculate TP, TN, FP, and FN. Table 10 and Table 11 shows how to calculate these values for the classes “!” and “R”. For the case of class “!” the TP are the instances located at the intersection between the row and the column of the same class; if we look at the confusion matrix in Table 9 the value of TP is 470. To calculate FP, we add all the values of the column cells where the class “!” is located In Table 9; we subtract the value of TP from the result, obtaining that FP is 2. FN is obtained by adding all the values of the row where the class “!” is located. In Table 9 we subtract the value of TP from this and obtain that FN is 0. Finally, to calculate TN, we add all the values of the cells of the confusion matrix and subtract TP, FP, and FN, and thus obtain that TN has a value for class “!” of 5221. If we apply the same procedure for class “R”, we observe that it has a TP value of 1802; by subtracting this value from the sum of the cells in the column we obtain that FP is equal to 0. Additionally, if we subtract the TP value from the sum of the cells in the row FN, it gets a value of 1. Finally, if the sum of all the cells in the confusion matrix, we subtract the TP, FP, and FN values, we obtain that TN is 3890.

Table 12 shows the TP, FP, FN, and TN values for the eight arrhythmia classes. We use these values and the equations presented in Section 4.5.1 to calculate the metric values F1 score, accuracy, sensitivity, specificity, and precision for each arrhythmia class. For example, to calculate the sensitivity value of class “E”, we have that TP = 106 and FN = 1 using these values and Equation (4) we obtain a value of 99.07%.

The values presented in Table 13 are the individual measures for each arrhythmia of the best solution presented in experiment 2, obtained with the above procedure and the equations of Section 4.5.1. The best values are obtained by class “a” (aberrant atrial premature beat), “N” (normal beat), and “V” (premature ventricular contraction beat). The other classes have performances between 99% and 100% in all metrics. It is essential to remark that the specificity and sensibility range is between 99.07% and 100% for all the classes. These results indicate that less than an 1% is the margin of error of the classifier for each individual arrhythmia.

### 5.2. Parametric Analysis of the Classification Model

In order to prove that the system reaches at least 98% of the F1 score, we carried out a parametric distribution analysis based on a T-Student test. We propose the next hypothesis:

***H*_0_:**  *The approach proposed obtains a value of F1 score lower than or equal to 98% in classification of eight classes of arrhythmias (N, L, R, V, !, j, a, E).*

***H*_1_:**  *The approach proposed obtains a value of F1 score higher than 98% in classification of eight classes of arrhythmias (N, L, R, V, !, j, a, E).*

**Figure 13:**
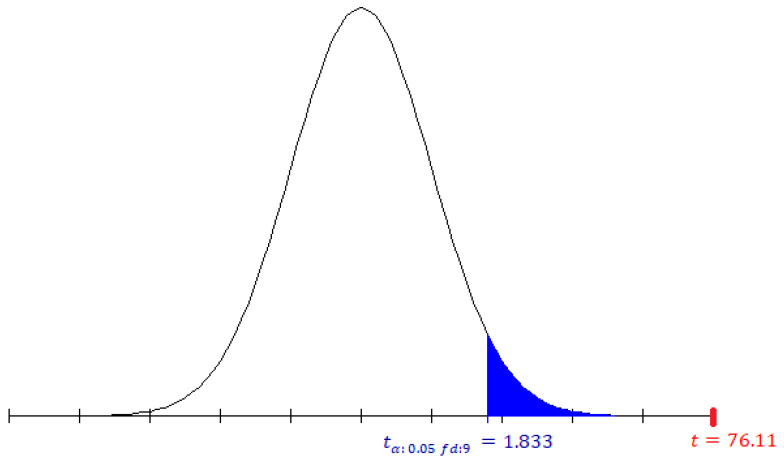
T-Student statistic from the approach proposed of experiment 2.

Using the experiment 2 results of Table 6, we have the following sample data: (a) mean x=99.77, (b) sample size n=10, (c) standard deviation S=0.073. By considering the degrees of freedom (df=9) and the significance level (α=0.05) for the parametric analysis, the value corresponding to the T-Student statistic is $t_{\alpha :0.05,fd:9}=1.833$. We use the Equation (8) and obtain t=76.11 of the Student T-value. Figure 13 shows the one-tailed test result of the previously calculated value, we observe that the Student T-value (76.11) is greater than T-Student statistic (1.833), for this reason, the null hypothesis (*H*_0_) is rejected, and the alternative hypothesis is accepted (*H*_1_), this gives evidence that the performance of our approach reaches values of F1 score greater than 98% in most tests.

(8) $$t=\frac{\bar{x}-\mu_{0}}{S/\sqrt{n}}=\frac{99.77-98}{0.073/\sqrt{10}}=76.11$$

## 6. Discussion

Table 14 shows the performance of our study versus works of state of the art. We select as the best solution the experiment 2, from analysis. The four metrics used to compare our best solution to other works are accuracy, sensitivity, precision, and F1 score; Section 4.5.1 explains the process to compute the metrics and the objective of each measure.

Hassanien et al. [10] and Sarvan and Ozkurt [12] depicted problems with the unbalanced dataset, obtaining a low performance in sensitivity with values of 45.49% and 26.85% respectively. Moreover, Hassanien et al. [10] obtained a 45.48% of F1 Score; the low value of this metric show that their classifier model had problems with the learning of minority classes. Ashtiyani et al. [11] used only three classes to train their model, and this is a number of classes lower than our work.

In the work of Rajesh & Dhuli [13], their dataset had lower unbalance compared with our study. We computed the imbalance ratios (IR) of classes and observed that the maximum value was 50.77 in their dataset against the maximum value of our work with an IR of 705.3. In Section 3.2, we point out that a high value of IR is related to a high unbalance, which can deteriorate the performance of the classifier. Jian et al. [14] in their study showed a high value of accuracy (98.4%). Still, they did not report the values of other metrics as Sensitivity nor F1 Score; this is a disadvantage because the performance of the classifier model is not visible when it learns about minority classes.

Gao et al. [15] classified a similar number of arrhythmias that our work and their dataset was unbalanced too, the classes used by the authors were: normal, left bundle branch block, right bundle branch block, atrial premature contraction, nodal escape beat, aberrant atrial premature beat, nodal (junctional) premature beat, and atrial escape beat. They utilized hold-out validation with 90% to training and 10% to testing, and implemented an LSTM Recurrent Network, obtaining a performance above 90%. Nevertheless, our work we exceed them in a 0.69% accuracy, sensitivity over 0.61%, in precision over 0.59%, and F1 score over 0.61%. This observation is important due to avoiding false positives and false negatives in the diagnosis, the risk of injuring the patient decreases. The hold-out validation is a method that divides a dataset into two subsets, one subset to train the classifier and the other to test the performance of the model; the disadvantage of the method is that the performance is more variable due to the small size of testing data. Therefore, we use a robust method called K fold cross-validation. It divides a dataset into K subsets or folds, the testing set is a subset, and the training set is the remaining subsets when each has been used as the testing set, the process stops. This method is a better indicator of the quality of the model because it allows us to observe the behavior of the model with unseen data, thus there is a tendency for less variation in the results due to the whole dataset is used to training and testing.

Additionally, it is important to remark that only two authors used a metaheuristic to optimize the feature selection stage in the classification process. Hassanien et al. [10] used a metaheuristic based on the behavior of elephants, and Ashtiyani et al. [11] implementing a genetic algorithm. The other authors mentioned in Table 14 optimize the classification process using parameters tuning. In this sense, Gao et al. [15] obtained the best performance. Comparing the works of Table 14, we conclude that the use of a metaheuristic allows us to carry out an extensive exploration of combinations of parameters, to improve the performance of the classifier model. This exploration would have been complicated using only parameters tunning (experience). Hence, our approach allows us to surpass the results obtained in each metric of the works reported in the state of the art.

Finally, we also have carried out some experiments based on the Association for the Advancement of Medical Instrumentation (AAMI), who suggests using five classes according to the standard: ANSI/AAMI EC57:1998 [30]. The fifteen heartbeat classes that have the MIT-BIH Arrhythmia Database are grouped into 5 superclasses: normal (N), supraventricular ectopic beat (SVEB), ventricular ectopic beat (VEB), fusion beat (F) and unknown beat (Q). These experiments have been considered to show the usefulness of the metaheuristic approaches presented considering other contexts. Table 15 shows that the results obtained by our approach differential evolution with a high-low pass filtering, 99.19% accuracy, and a 98.79% of sensibility, compared to Rajesh and Dhuli [13] and Sarvan and Ozkurt [12] have obtained a better performance.

There are a variety of methods to extract characteristics, such as statistical, morphological, QRS complex, and Wavelet characteristics [30]. Under different circumstances and contexts, their election affects the results when identifying cardiac abnormalities. Among this variety, each author can select a different window (signal range) to extract characteristics in the time domain, the frequency domain, or the time-frequency domain. Its choice depends directly on the author’s experience and the algorithms used for its classification. Each of these methods presents advantages and disadvantages when they are used to classify arrhythmias. Therefore, determining whether it is performed by segments of eight seconds, dynamic time intervals, or by beat, such as it is our case, is an open problem today.

If we want to answer this problem, we must compare the proposals under the same conditions using a standard that allows a fairer comparison between them. One of these parameters is the ANSI/AAMI standard, since it allows a fair comparison among different methods [48]. Most of the studies do not present this type of comparison, which makes it challenging to answer throughout the state-of-the-art review in a cross-sectional way. Besides, authors do not contemplate the same arrhythmia numbers or classes, such as [10,14] with 2, [11] with 3, [12,13] with 5, [15], and our work with 8. In our work, we have included some comparisons using ANSI/AAMI standard to compare our approach with Sarvan and Ozkurt [12] and Rajesh and Dhuli [13] showing better results.

On the other hand, when we can not make a fair comparison, authors could claim that their work presents competitive results, such as the work of Hajeb-Mohammadalipour et al. [19] or our work. Nevertheless, the datasets and the results reported can not be compared directly because of the lack of a database standardized such as ANSI/AAMI.

As a remark, we consider that our proposal contributes positively to arrhythmia classification from unbalanced data due to the fact that the arrhythmias classification is carried out by beat analysis with competitive and balanced results; this means that we obtain an accuracy, sensitivity, specificity, precision, and F1 score with less than 1% of error and some of them with a 0% of error as we show in the individual and overall analysis of the proposal.

## 7. Conclusions

In electrocardiogram classification handling unbalanced data, the optimization parameters of the classifier play a key role because the performance depends on it. Hence, we present a metaheuristic optimization approach by combining undersampling based on the clustering method (data level), and a feature selection method (algorithmic level). We selected an unbalanced subset to classify seven types of arrhythmias and a normal beat: left bundle branch block beat, right bundle branch block beat, premature ventricular contraction beat, ventricular flutter wave beat, nodal (junctional) escape beat, aberrated atrial premature beat, ventricular escape beat, and normal beat. We have compared two metaheuristic approaches based on a differential evolution and a particle swarm optimization applying two filtering methods. We had a higher accuracy when we combined a high-pass and low-pass filtering method and a differential evolution as a process to optimize an artificial neural network. The values of the metrics obtained showed an accuracy of 99.95%, an F1 score of 99.88%, a sensitivity of 99.87%, a specificity of 99.99%, and a precision of 99.89%. Hence, our results surpass the metrics reported in the state of the art. Our proposed models can assist expert clinicians and cardiologists in accurately detecting arrhythmias, after developing a routine ECG test, in order to save time and decrease the number of misdiagnoses. Furthermore, with the models proposed along with low-cost ECG devices, we are looking for widespread use of the ECG as a diagnostic tool in remote places where access to a cardiologist is not possible. In future works, we are going to focus on testing our approach with other unbalanced databases, and improving the computational costs related to metaheuristic search. Additionally, we are interested in extending our work by introducing other classes of arrhythmia beats and classes of arrhythmia of the rhythm type.

## Figures and Tables

**Table 1:** Classes selected of MIT-BIH Arrhythmia Database.

Class	Name	Number of Samples
N	Normal beat	74,758
L	Left bundle branch block beat	8072
R	Right bundle branch block beat	7255
V	Premature ventricular contraction beat	7123
!	Ventricular flutter wave beat	472
j	Nodal (junctional) escape beat	229
a	Aberrated atrial premature beat	150
E	Ventricular escape beat	106

**Table 2:** IR values between majority and minority classes.

		Majority Classes			
		N	L	R	V
**Minority classes**	!	158.4	17.1	15.4	15.1
	j	326.5	35.2	31.7	31.1
	a	498.4	53.8	48.4	47.5
	E	705.3	76.2	68.4	67.2

**Table 3:** Comparison test to select the number of subsegments. Abbreviations: HL: combination of High-pass and low-pass filtering; DE: differential evolution.

	DE-HL 50 Features (10 Subsegments)		DE-HL 25 Features (5 Subsegments)	
	Accuracy	F1 Score	Accuracy	F1 Score
**Mean**	99.92	99.77	99.92	99.76
**Standard Deviation**	0.032	0.073	0.047	0.094

**Table 4:** Representation vector. Abbreviations: SOM: self organizing map.

1	2	3	4	5	6	7	...	56
SOM	Class	Class	Class	Class	Number	Features		
map	L	N	V	R	of	to		
size	Percentage	Percentage	Percentage	Percentage	neurons	select		

**Table 5:** Experimental configuration. Abbreviations: HL: combination of high-pass and low-pass filtering; PSO: particle swarm optimization; n: population; $w_{max}$: maximum inertia weight; $w_{min}$: minimum inertia weight; $c_{1}$: accelerate constant 1; $c_{2}$: accelerate constant 2; DE: differential evolution; Cr: crossover rate, F: mutation factor.

	Experiment 1	Experiment 2	Experiment 3	Experiment 4
**Filtering Configuration**	HL Filtering	HL Filtering	Wavelet Filtering	Wavelet Filtering
**Metaheuristic Configuration**	**PSO:** n = 50 $w_{max}$ = 0.9 $w_{min}$ = 0.4 $c_{1}$ = 2 $c_{2}$ = 2 Evaluations = 2000	**DE:** n = 50 Cr = 0.9 F = 0.5 Evaluations = 2000	**PSO:** n = 50 $w_{max}$ = 0.9 $w_{min}$ = 0.4 $c_{1}$ = 2 $c_{2}$ = 2 Evaluations = 2000	**DE:** n = 50 Cr = 0.9 F = 0.5 Evaluations= 2000
**Bounds**	Map Size: 2–10 L %: 0.01–0.999 N %: 0.001–0.999 R %: 0.01–0.999 V %: 0.01–0.999 No. Neurons: 10–500 Features: 0–1	Map Size: 2–10 L %: 0.01–0.999 N %: 0.001–0.999 R %: 0.01–0.999 V %: 0.01–0.999 No. Neurons: 10–500 Features: 0–1	Map Size: 2–10 L %: 0.01–0.999 N %: 0.001–0.999 R %: 0.01–0.999 V %: 0.01–0.999 No. Neurons: 10–500 Features: 0–1	Map Size: 2–10 L %: 0.01–0.999 N %: 0.001–0.999 R %: 0.01–0.999 V %: 0.01–0.999 No. Neurons: 10–500 Features: 0–1

**Table 6:** Experiments results of ten tests in percentages. Abbreviations. SD: standard deviation.

	Experiment 1	Experiment 2	Experiment 3	Experiment 4
**Test**	**F1 Score**	**F1 Score**	**F1 Score**	**F1 Score**
**1**	99.74	99.74	99.50	**99.69**
**2**	99.84	99.70	99.56	99.47
**3**	99.73	99.74	99.30	99.61
**4**	99.76	99.73	99.51	99.68
**5**	99.82	99.76	99.65	99.52
**6**	99.61	99.65	99.27	99.56
**7**	99.26	**99.88**	99.62	99.54
**8**	**99.93**	99.78	99.14	99.68
**9**	99.62	99.87	**99.81**	99.66
**10**	99.41	99.83	99.62	99.66
**Mean**	99.67	99.77	99.50	99.61
**SD**	0.205	0.073	0.204	0.079

**Table 7:** Best solutions of the experiments on ten tests.

	Experiment 1	Experiment 2	Experiment 3	Experiment 4
**F1 Score**	99.93 %	99.88 %	99.81 %	99.69 %
**Accuracy**	99.95 %	99.95 %	99.94 %	99.88 %
**Sensitivity**	99.96 %	99.87 %	99.78 %	99.85 %
**Specificity**	99.99 %	99.99 %	99.99 %	99.98 %
**Precision**	99.89 %	99.89 %	99.83 %	99.53 %
**SOM Map Size**	2	2	2	4
**Instances of a**	150	150	150	150
**Instances of E**	106	106	106	106
**Instances of j**	229	229	229	229
**Instances of !**	472	472	472	472
**Instances of L**	4272	2784	3299	1269
**Instances of N**	113	77	159	2160
**Instances of V**	817	73	300	921
**Instances of R**	2167	1802	1887	2463
**Size of the new subset**	8326	5693	6602	7770
**Number of neurons**	207	500	126	86
**Amount of Features selected**	23	44	27	43
**Features Selected**	2-4,8,9,14,17,24–26, 28,31,32,34,36,40,41, 44–47,49	1–5,7–13,15,17, 19-23, 25–29,31–50	1,6,8,11,12,14–18, 20,21,25,28,29, 31–35,37–39,41, 44,46,48	1–5,7–9,11,12,14–18, 21–27,29-31,33–50

**Table 8:** redIR values comparison between full dataset and dataset of the best solution on experiment 2.

		Majority Classes							
		**Full Dataset**				**Experiment-2 Dataset**			
		**N**	**L**	**V**	**R**	**N**	**L**	**V**	**R**
**Minority classes**	**!**	158.4	17.1	15.4	15.1	0.2	5.9	3.8	0.2
	**j**	326.5	35.2	31.7	31.1	0.3	12.2	7.9	0.3
	**a**	498.4	53.8	48.4	47.5	0.5	18.6	12.0	0.5
	**E**	705.3	76.2	68.4	67.2	0.7	26.3	17.0	0.7

**Table 9:** Confusion matrix obtained by the best solution of experiment 2.

	Predicted Label								
		**a**	**E**	**j**	**!**	**L**	**N**	**V**	**R**
**True** **Label**	**a**	150	0	0	0	0	0	0	0
	**E**	0	106	0	1	0	0	0	0
	**j**	0	0	228	0	0	0	0	0
	**!**	0	0	0	470	0	0	0	0
	**L**	0	0	0	1	2784	0	0	0
	**N**	0	0	0	0	0	77	0	0
	**V**	0	0	0	0	0	0	73	0
	**R**	0	0	1	0	0	0	0	1802

**Table 10:** Examples of how to compute TP, TN, FP, and FN of class “!”.

	a	E	j	!	L	N	V	R
**a**	**TN**			**FP**	**TN**			
**E**								
**j**								
**!**	**FN**			**TP**	**FN**			
**L**	**TN**			**FP**	**TN**			
**N**								
**V**								
**R**								

**Table 11:** Examples of how to compute TP, TN, FP, and FN of class “R”.

	a	E	j	!	L	N	V	R
**a**	**TN**							**FP**
**E**								
**j**								
**!**								
**L**								
**N**								
**V**								
**R**	**FN**							**TP**

**Table 12:** Results of TP, TN, FP, and FN from confusion matrix of Table 9.

	a	E	j	!	L	N	V	R
**TP**	150	106	228	470	2784	77	73	1802
**FP**	0	0	1	2	0	0	0	0
**FN**	0	1	0	0	1	0	0	1
**TN**	5543	5586	5464	5221	2908	5616	5620	3890

**Table 13:** Performance of the best solution on experiment 2 in percentage.

	Class	a	E	j	!	L	N	V	R
Metric									
**F1 Score**		100	99.53	99.78	99.79	99.98	100	100	99.97
**Accuracy**		100	99.98	99.98	99.96	99.98	100	100	99.98
**Sensitivity**		100	99.07	100	100	99.96	100	100	99.94
**Specificity**		100	100	99.98	99.96	100	100	100	100
**Precision**		100	100	99.56	99.58	100	100	100	100

**Table 14:** Comparison of the method proposed with the state of the art.

	Accuracy	Sensitivity	Precision	F1 Score	No. Arrhythmias
**Hassanien et al. [10]**	93.31	45.49	46.45	45.48	2
**Ashtiyani et al. [11]**	97.14	97.54	97.64	—-	3
**Rajesh & Dhuli [13]**	99.1	97.9	—-	—-	5
**Sarvan & Ozkurt [12]**	93.72	26.85	85.43	—-	5
**Jiang et al. [14]**	98.4	—-	—-	—-	2
**Gao et al. [15]**	99.26	99.26	99.30	99.27	8
**Proposed**	99.95	99.87	99.89	99.88	8

**Table 15:** Comparison of the method proposed with other works using the standard AAMI.

AAMI Standard				
	**Accuracy**	**Sensitivity**	**Precision**	**F1 Score**
**Rajesh & Dhuli [13]**	99.1	97.9	—	—
**Sarvan & Ozkurt [12]**	93.72	26.85	85.43	—
**Proposed**	99.19	98.79	98.80	98.79

## Abbreviations

The following abbreviations are used in this manuscript:

AAMI	Association for the Advancement of Medical Instrumentation
ANN	Artificial Neural Network
ANSI	American National Standards Institute
BPM	Beats Per Minute
CNN	Convolutional Neural Network
DE	Differential Evolution
DWT	Discrete Wavelet Transform
ECG	Electrocardiogram
EHO	Elephant Herding Optimization
FN	False Negatives
FP	False Positives
Hz	Hertz
IR	Imbalance Ratio
LSTM	Long Short-Term Memory
MMNNS	Multi-Module Neural Network System
MPTA	Modified Pan–Tompkins Algorithm
PSO	Particle Swarm Optimization
RUS	Random Under-Sampling
SMOTE	Synthetic Minority Oversampling Technique
SOM	Self-Organizing Map
SVM	Support Vector Machine
TN	True Negatives
TP	True Positives
